# 2-MCPD-Induced Effects in the Heart: Toxicological and Mechanistic Implications from Comparative Proteomic Analyses in Rats

**DOI:** 10.3390/molecules31040692

**Published:** 2026-02-17

**Authors:** Axel Oberemm, Andreas Eisenreich, Katharina Sommerkorn, Anna Reinhold, Christine Meckert, Mario E. Götz

**Affiliations:** German Federal Institute for Risk Assessment, Department of Food and Feed Safety in the Food Chain, Max-Dohrn-Straße 8-10, 10589 Berlin, Germanymario.goetz@bfr.bund.de (M.E.G.)

**Keywords:** proteomics, heart, toxicology

## Abstract

The toxic actions of 2-monochloropropane-1,3-diol (2-MCPD) are still less well understood than those of 3-monochloropropane-1,2-diol (3-MCPD). The toxic effects of 2-MCPD on the heart, especially at the proteomic level, were recently investigated by researchers in a subacute (28 days, in 2017) and in a subchronic (90 days, in 2024) oral toxicity rat study. Here, we set out to perform an updated analysis and re-evaluation of these proteomic in vivo data in a comparative manner and in the context of 2-MCPD metabolism, focusing in particular on mitochondrial energy metabolism and the maintenance of the structural integrity and function of the heart. The aim of our project was to develop further reasonable, toxicologically relevant research hypotheses for future studies addressing this topic in order to shed more light on the—so far—rather limited knowledge of the toxicological properties and modes of action of 2-MCPD. Our updated data analysis and comparative re-evaluation revealed strong indications of cytoskeletal protein deregulation, indicative of cardiomyocyte degeneration, and the deregulation of enzyme proteoforms linked to carbohydrate utilization and mitochondrial functions. This led us to hypothesize that reactive metabolites of 2-MCPD, other than those formed from 3-MCPD, could impair mitochondrial pyruvate utilization and mitochondrial energy production, potentially resulting in cardiac functional heart failure in rats at doses slightly higher than 10 mg 2-MCPD per kg bw/day. Thus, we postulate the intermediate formation of some putative aldehydic and acidic metabolites following oral 2-MCPD exposure that might be causative of cardiotoxicity in rats.

## 1. Introduction

2-Monochloropropane-1,3-diol (2-MCPD) and its esters are heat-induced food contaminants that belong to the group of chloropropanols [1]. 2-MCPD and its esters, as well as comparable compounds, such as 3-monochloropropane-1,2-diol (3-MCPD) and its esters, are chlorinated glycerin-derived fatty acid esters, which are predominantly generated during refinement of plant-based fats and oils in a process called “deodorization”, in which unwanted odors and flavors are removed at high temperatures [2]. These compounds are detected in many food categories, such as hydrolyzed vegetable proteins, baked cereal products, infant formula, and refined vegetable oils [3].

Based on the available data, it is assumed that 3-MCPD esters are almost completely cleaved in the digestive tract and that only free 3-MCPD is present in blood after oral application [4]. This is also assumed for 2-MCPD, which is substantiated by in vitro data obtained by Kaze et al. in 2016, showing that di- and monooleate of 2-MCPD were hydrolyzed by pancreatic lipase and pancreatin in Caco2 cells [5]. This setting was used as a model for intestinal digestion and absorption. The presented data indicate that 2-MCPD fatty acid esters are hydrolyzed in the digestive tract to free 2-MCPD, which is then absorbed into the bloodstream [5].

3-MCPD has been found to induce nephrotoxic effects (e.g., renal tubular hyperplasia) and reproductive toxicity (i.e., male antifertility effects), as well as non-genotoxic carcinogenic effects (e.g., renal tubular tumors) in rodents [6,7,8]. Based on the available toxicological data, the potential risk of 3-MCPD and its esters to human health has been assessed by risk assessment authorities, including the European Food Safety Authority (EFSA) [4]. Considering the available data, EFSA derived a tolerable daily intake of 2 μg/kg body weight (bw) per day (d) for free and ester-bound 3-MCPD as a health-based guidance value (HBGV) in 2018 [9]. Consequently, legal restrictions were put in place in the European Union (EU) to minimize consumer exposures towards free and ester-bound 3-MCPD in various food matrices (Commission Regulation (EU) 2023/915; https://eur-lex.europa.eu/eli/reg/2023/915/oj/eng, accessed on 5 September 2025).

In contrast to 3-MCPD, the amount of toxicological data on 2-MCPD and its esters is very limited. Therefore, no HBGV has been established for 2-MCPD and its esters due to insufficient toxicological information, such as missing data on metabolism and long-term toxicity, as well as limited data on its modes of action [3]. Due to these limitations, 2-MCPD and its esters are so far not regulated in the European Union.

The available toxicological data indicate that the most sensitive target organs for the toxic actions of 2-MCPD are most probably not the same as those of 3-MCPD. This also applies to the underlying putative pathogenic mechanisms induced by these substances [10,11].

Kidneys and testes were identified as the most sensitive target organs of 3-MCPD after oral treatment of rodents [3,9]. It is known that 3-MCPD inhibits glycolytic enzymes, particularly in the Leydig cells and spermatocytes of rodents, which might account for the observed adverse effects in the rodent testis [12,13]. Together with earlier studies demonstrating that metabolites of 3-MCPD inhibit glycolysis [14,15,16], this data indicates that glycolytic energy utilization is amongst the key molecular targets that elicit 3-MCPD-mediated modes of action. The nephrotoxic effects observed in rodents after oral exposure to 3-MCPD are most probably induced by oxidative metabolites of 3-MCPD, finally leading to oxalic acid, a well-known nephrotoxic compound [4,6,17,18].

In contrast to 3-MCPD, 2-MCPD showed only minor effects in the kidneys and testes, determined via oxidative stress induced by oral exposure of mice to 2-MCPD for 28 days [10,11]. Results of other studies have indicated that the heart is a major target organ for the toxic effects induced by 2-MCPD [19,20]. Cardiotoxic effects of 2-MCPD were already demonstrated in a 28-day rodent study performed by Perrin et al. in 1994 (unpublished report No. RE-SR94026, reviewed in [3]). In this study, 10–15 male and female Sprague–Dawley rats were orally exposed by gavage to 2, 16 or 30 mg 2-MCPD/kg body weight (bw)/day. The authors described that some rats died in the high-dose group (30 mg/kg bw/day), which was attributed to cardiac failure. In this context, different pathophysiologically relevant effects were observed in 2-MCPD-treated animals, including dose-dependent lesions in striated muscle, cytoplasmic vacuolization, and myocyte lysis in the middle- and high-dose groups (16 and 30 mg/kg bw/day) at days 8 and 29. These effects were found to be most extensive and severe in the myocardium. Moreover, over-contraction of the heart was observed at autopsy. These effects were accompanied by altered clinical chemistry parameters, such as increased levels of aspartate aminotransferase (ASAT), alanine aminotransferase (ALAT), lactate dehydrogenase (LDH), and creatine kinase (CK), as well as increased phosphorus and potassium values. Finally, functional cardiac adaptation was observed in less severely affected animals at day 29 [3].

Substantiating these findings, cardiotoxic effects of 2-MCPD were also shown in a 90-day oral exposure study performed with Fischer F344/CDF rats in 2018 [20]. In this study, rats were treated for 90 days with 0 to 40 mg 2-MCPD/kg bw/day. Compared to controls, 40 mg 2-MCPD/kg bw/day led to elevated heart weights and induced cardiac lesions characterized as multifocal myocardial and interstitial vacuolation as well as fibrosis and necrosis. Besides this histopathological characterization, proteomic analyses were also done with hearts isolated from male Fischer F344/CDF rats orally exposed to 0, 2, 10, or 40 mg 2-MCPD/kg bw/day in this study. Based on their findings, the authors hypothesized that 2-MCPD mediates cardiotoxic effects in rats through inflammatory activation and suppression of metabolic and cardiac functions [20]. Moreover, this data indicates that some mitochondrial functions might be predominantly compromised selectively by 2-MCPD metabolites in differentiated and non-proliferating cells [20]. However, neither the molecular targets of 2-MCPD-metabolites nor the putative mechanisms of impairment of mitochondrial energy metabolism have been discussed in this context.

Based on the cardiotoxic effects observed in the abovementioned 28-day study by Perrin et al., 1994 (reviewed in [3]), a 28-day oral toxicity study with male Wistar rats orally exposed by gavage to 10 mg/kg bw/day 2-MCPD or 3-MCPD was done in 2016 to further characterize the effects of 2-MCPD on the heart [21]. In this context, proteomic analyses were also performed with hearts isolated from these rats after 28 days [19].

However, the underlying modes of action of 2-MCPD in the heart are—so far—unknown [4,22].

Here, we set out to identify potential modes of action of 2-MCPD in the heart involving impairment of energy metabolism. In this context, we performed an updated analysis and comparative re-evaluation of the proteomic data derived from two rat studies that used 2-MCPD solely as a toxic compound orally exposed to rats [19,20]. Based on this data, we investigated the potential modes of action, taking into account relevant metabolic implications, and developed/postulated working hypotheses for future projects of high relevance regarding the toxicological evaluation of 2-MCPD and its esters in food.

## 2. Results

### 2.1. Animal Studies

In both studies, no signs of overt toxicity were observed in the hearts at 10 mg/kg bw/day. However, in all animals of the 40 mg/kg bw/day dose group from the 90-day study, histopathological changes were identified in the hearts, including myofiber and interstitial vacuolation with multifocal necrosis [20]. More detailed information can be found in [19,20].

In the following part, we will describe the results of a new post hoc and updated analysis of the proteomic data obtained from the two abovementioned oral toxicity studies in rats treated with 2-MCPD [19,20]. Technical details of the studies are described in Section 4.1.

### 2.2. Proteomic Analysis of Differentially Expressed Proteins

In total, 143 protein spots (115 upregulated, 28 downregulated) were detected in the subacute study (28 days) and 46 spots (26 upregulated, 20 downregulated) in the subchronic study (90 days) at 10 mg/kg bw/day 2-MCPD. Interestingly, upregulation was more prevalent than downregulation in both studies, but in the 28-day study, this outcome was much more pronounced. Further counts were 29 spots (11 upregulated, 18 downregulated) at 2 mg/kg bw/day and 198 spots (163 upregulated, 35 downregulated) at 40 mg/kg bw/day in the subacute study, where more dose groups were available. Remarkably, the number of spots detected in the highest treatment group of 40 mg/kg bw/day in the 90-day study was similar to the number of deregulated spots found in the 28-day study at 10 mg/kg bw/day. Out of 143 spots, 113 proteins were identified by mass spectrometric analysis in the 28-day study (79%), and out of 238 spots, 203 proteins were identified in the 90-day study (85%). Roughly half of the number of deregulated unique proteins—48 out of 85 found in the subacute treatment at 10 mg/kg bw/day—were consistently detected in both studies. The greatest overlap of 39 proteins was shown between the 10 mg/kg bw/day group of the 28-day study and the 40 mg group of the 90-day study. An unusually high number of proteins were detected in different protein spots, indicating different proteoforms of the same protein. This finding was much more pronounced in the subchronic approach, where 36 proteins were present in 110 modifications compared to 11 proteins in 38 modifications in the subacute study (for a complete list of identified protein spots in all dose groups, refer to Supplemental Table S2).

### 2.3. Ingenuity Pathway Analysis (IPA)

#### 2.3.1. Toxicity Functions and Categories

**Figure 1:**
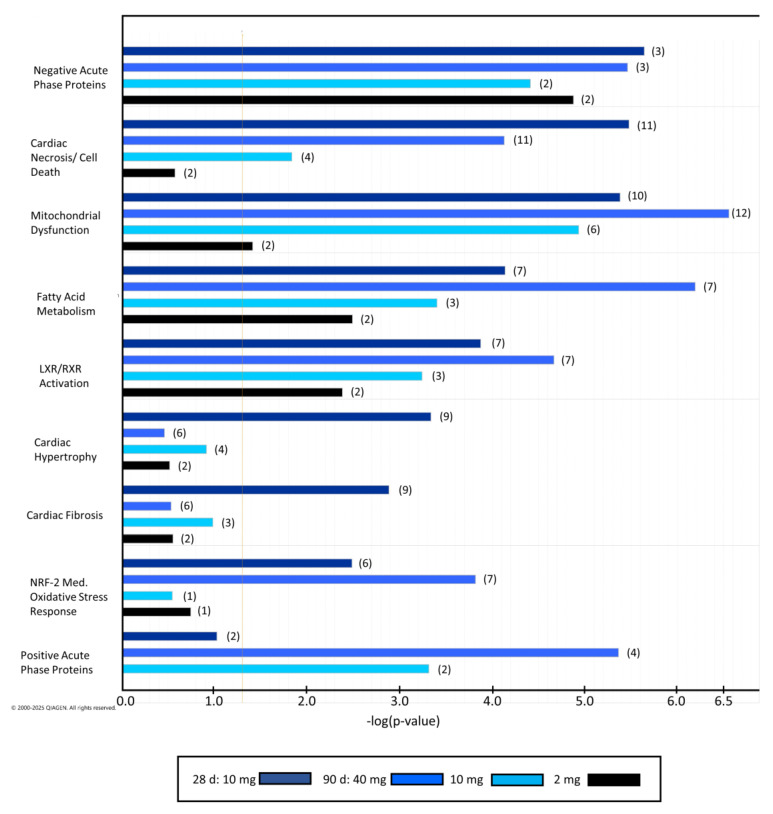
Results from Ingenuity Pathway Analysis: selected toxicity categories and mechanisms found in rat heart based on patterns of deregulated proteins following oral exposure of 2-MCPD after 28 days (10 mg/kg bw/day) and 90 days (2, 10, 40 mg/kg bw/day). The results of the 28-day study are depicted in comparison to the results of the 90-day study (in descending dose order). Numbers in brackets indicate the number of proteins related to endpoints. More details and protein identities are provided in Table S3.

As already shown by the comparison of deregulated spot numbers, effects highlighted by IPA were most pronounced in the high-dose groups of the 28-day study at 10 mg/kg bw/day and of the 90-day study at 40 mg/kg bw/day. This is reflected in the IPA-predicted toxicity functions and categories of deregulated proteins (Figure 1), showing the highest significance of the aforementioned treatments. Remarkably, many categories were also significant in the low-dose group of the 90-day study at 2 mg/kg bw/day. Most of the effects were related to heart and mitochondrial toxicity, but associations with negative acute phase response proteins and fatty acid metabolism were also found. No relations to positive acute phase response proteins were obtained for the 28-day study. For a complete list of toxicity categories and details of deregulated proteins, refer to Supplemental Table S3.

#### 2.3.2. Related Pathways

In both high-dose treatments of the 28-day and 90-day studies at 10 and 40 mg/kg bw/day, respectively, deregulated proteins were found to be related to pathways of mitochondrial protein degradation. This finding is based on the deregulation of acetyl-CoA acetyltransferase (Acat1), aconitate hydratase (Aco2), ATP synthase subunit alpha (Atp5f1a), ATP synthase subunit beta (ATP5f1b), delta(3,5)-delta(2,4)-dienoyl-CoA isomerase (Ech1), glutamate dehydrogenase 1 (Glud1), 2-oxoglutarate dehydrogenase complex component E1 (Ogdh), succinyl-CoA:3-ketoacid coenzyme A transferase 1 (Oxct1), pyruvate dehydrogenase E1 component subunit alpha (Pdha1), and pyruvate dehydrogenase (acetyl-transferring) kinase isozyme 1 (Pdk1) in the 28-day study at 10 mg/kg bw/day and acetyl-CoA acetyltransferase (Acat1), aldehyde dehydrogenase (ALDH2), ATP synthase subunit alpha (Atp5f1a), ATP synthase subunit beta (Atp5f1b), ATP synthase subunit gamma (Atp5f1c), dihydrolipoyl dehydrogenase (Dld), delta(3,5)-delta(2,4)-dienoyl-CoA isomerase (Ech1), enoyl-CoA delta isomerase 1 (Eci1), fumarate hydratase (Fh), trifunctional enzyme subunit alpha (Hadha), succinyl-CoA:3-ketoacid coenzyme A transferase 1 (Oxtc1), and pyruvate dehydrogenase E1 component subunit alpha (Pdha1) in the 90-day study at 40 mg/kg bw/day (Table 1). Also, at 10 and 2 mg/kg bw/day in the 90-day study, some of these proteins appeared deregulated. Remarkably, most of the aforementioned deregulated proteins were present after subacute (28-day) as well as after subchronic (90-day) treatment. For the complete list of canonical pathways and associated patterns of deregulated proteins, refer to Supplemental Table S4.

#### 2.3.3. Network Analysis

The network analysis presented here for each 2-MCPD dose (see Supplemental Figure S1) is based on the results of the proteomic analyses conducted in the 28- and 90-day studies by Schultrich et al. and Cayer and colleagues, respectively [19,20]. The resulting systems represent a hypothetical interaction network for 2-MCPD-induced effects in rats, allowing the identification of candidates potentially involved in the regulation and/or mediation of 2-MCPD effects. In this context, the analyses predict potential relationships between different factors (e.g., transcription regulators, kinases, transporters, etc.), linked via downstream activation (red lines) or inhibition (blue lines), as well as provide information on findings inconsistent with the state of the downstream molecule (yellow lines) or unpredicted effects (black lines). Networks for all dose groups are available in Supplemental Figure S1.

The most obvious and interesting result was found when comparing the network analyses for 2 and 10 mg 2-MCPD/kg bw/day of the 90-day study (Supplemental Figure S1). In these dose groups, the upstream regulator p53 was predicted by IPA as a highly ranked regulator in networks related to cardiac damage, oxidative stress, and mitochondrial dysfunction. Interestingly, upregulation was predicted in the low-dose group at 2 mg/kg bw/day, whilst downregulation was forecasted in the mid-dose group at 10 mg/kg bw/day (Supplemental Figure S1). In this context, upregulation of mitochondrial cytoplasmic aconitate hydratase (Aco 1, Table S1) was correlated with increased p53 in only the network of 2 mg/kg bw/day. Moreover, downregulation of p53 at 10 mg/kg bw/day was associated with downregulation of Parkinson’s disease protein 7 (PARK 7, Table S1). These data indicate that p53 could play an important role in the regulation of the toxic effects of 2-MCPD in rat hearts. In networks of the remaining dose groups, no such predictions of the upstream regulator p53 exist. Further details can be found in Supplemental Figure S1.

## 3. Discussion

### 3.1. Considerations on Possible Modes of Action of 2-MCPD Regarding Cardiac Stress Response and Maintenance of Cardiac Structural Integrity and Function

When analyzing proteomic data obtained from the 28-day rat study, it can be seen that a majority of the proteins significantly deregulated after oral exposure to 2-MCPD are involved in the cardiac stress response or in the maintenance of the structural integrity and function of the heart. This is in line with earlier findings of other researchers, indicating that treatment with 2-MCPD leads to lesions as well as structural changes in the heart and affects the cardiac function [3,20,22].

Proteomic results obtained from the 28-day oral toxicity study [19] showed that exposure of Wistar rats to 10 mg 2-MCPD/kg bw/day led to an increased level of heat shock protein beta-1 (Hspb1, Table 1). In the context of cardiac stress response, induction of Hspb1 was shown to mediate protective effects following myocardial infarction in rats by contributing to the preservation of mitochondrial function, consequently leading to an improvement of cardiac contractile function [23]. This is in line with data showing an increased level of Hspb1 protein to protect chicken myocardial cells from acute heat stress in vitro [24]. Thus, increased expression of Hspb1 in the 28-day oral toxicity study may represent a compensatory response in the hearts of rats exposed to cardiotoxic 2-MCPD. Substantiating this, an elevated cardiac level of Hspb1 was also found in Fischer F344/CDF rats exposed to 40 mg 2-MCPD/kg bw/day in the 90-day oral toxicity study (Table 1). Besides this, other members of the heat shock protein family, including heat shock protein HSP 90-beta (Hsp90b1), heat shock 70 kDa protein 1A (Hspa1a), and endoplasmic reticulum chaperone BiP (Hspa5), were also increased in rats treated with 40 mg 2-MCPD/kg bw/day after 90 days. Furthermore, a dose-dependent increase in heat shock 70 kDa protein 4 (Hspa4) was observed in rats exposed to 10 or 40 mg 2-MCPD/kg bw/day. These proteins are also known to mediate cardioprotective effects, e.g., as chaperones or via interaction with other effector proteins [25,26,27,28,29]. Therefore, this data may indicate a potential compensatory mechanism in the heart of rats exposed to 2-MCPD, counteracting the cardiotoxic influence of 2-MCPD via elevating levels of cardioprotective factors, such as chaperones in the heart.

The cardiotoxic effect of 2-MCPD (10 mg/kg bw/day) in Wistar rats after 28 days of exposure is reflected by a significant reduction in factors involved in the structural integrity of the heart and cardiac functions, such as actin and alpha cardiac muscle 1 (Actc1). In line with this, proteins involved in myocardial structure and functions, such as Actc1 or myosin regulatory light chain 2 (Myl2), were also found to be reduced in the 90-day oral toxicity study done with Fischer F344/CDF rats at 10 or 40 mg 2-MCPD/kg bw/day (Table 1). Moreover, aortic smooth muscle actin (Acta2) was found to be deregulated in multiple isoforms in all treatment groups (Table S1, Supplemental Figure S1).

Actc1 is known to play an important role in maintaining the structural integrity of the myocardium and in cardiac remodeling [30,31]. Reduced expression of Actc1 was shown to be associated with the development of dilated cardiomyopathy (DCM) in an inducible cardiac-specific serum response factor (Srf) gene disruption mouse model in 2020 [32]. Moreover, transgenic overexpression of actin, alpha cardiac muscle 1, in this mouse model preserved cytoarchitecture from adverse cardiac remodeling during DCM development [31]. Myl2 was demonstrated to be required for thick-filament stabilization and contractility in the heart [33]. Loss of the ventricular isoform of Myl2 in a transgenic mouse model led to the development of embryogenic cardiomyopathy, and reduced Myl2 protein levels in ventricles were shown to be associated with the occurrence of idiopathic dilated cardiomyopathy in humans [34].

This data suggests that 2-MCPD-induced cardiotoxicity, as expressed by cardiac cell vacuolization, myocyte lysis, and the development of severe myopathy and heart failure in rats [3,20], may be mediated via reduction in structural and functional cardiac proteins, such as Actc1 and Myl2.

On the other hand, cardiac levels of other proteins involved in maintenance of structural and functional integrity of the heart, such as caveolin-3 (Cav3), macrophage-capping protein (Capg), desmin (Des), WD repeat-containing protein 1 (Wdr1), tropomyosin alpha-1 chain (Tpm1), tropomyosin beta chain (Tpm2), and tropomyosin alpha-3 chain (Tpm3)**,** were found to be increased in the 28-day oral toxicity study done with Wistar rats exposed to 10 mg 2-MCPD/kg bw/day.

These proteins were shown to play a role in cardiac pathologies. In this context, reduced expression of Cav3 has been observed in several experimental models of heart failure [35]. Cardiac overexpression of this protein was found to mediate protective effects in the context of cardiac hypertrophy, heart failure and cardiomyopathy [36,37]. Moreover, Wang et al. demonstrated in 2013 that Capg is involved in fibrosis in the post-ischemic heart of rats and that reduced generation of this protein led to inhibited activation of primary rat ventricular fibroblasts and collagen deposition in these cells [38]. Des is an integral component of cardiomyocytes and plays a central role in maintaining the structure of striated muscle cells and their cytoskeletal organization, and mutation of the Des-encoding gene can cause cardiomyopathies [39]. Substantiating this, development of progressive myopathy and cardiomyopathy was also shown experimentally in Des knockout mice, and transgenic recovery of the Des expression ameliorated cardiomyopathy in these mice [40]. Wdr1 is involved in heart development as well as in the growth and maintenance of cardiomyocytes [41]. In 2019, Huang et al. showed that adult cardiomyocyte-specific deletion of the Wdr1-encoding gene in mice led to the development of cardiac hypertrophy and impaired cardiac function in adult mouse hearts [42]. Tpm1, Tpm2, and Tpm3 chains are crucial for actin regulation and stability, and they play—amongst other things—an essential role in the regulation of muscle contraction, e.g., in the heart [43]. Moreover, Tpm1 was shown to be involved in heart development [44]. Furthermore, various mutations of cardiac tropomyosins were found to be associated with cardiomyopathies [45]. Moreover, overexpression of the Tpm1 chain was shown to stabilize actin filaments in muscle cells, and increased expression of the Tpm3 chain was demonstrated to protect cardiomyocytes against hypoxia-induced injury in the context of ischemic heart disease, underlining the function-maintaining role of tropomyosins [46,47].

The aforementioned data suggest that increased expression of Cav3, Capg, Des, Wdr1, and tropomyosins may mediate protective as well as structure- and function-preserving effects under pathophysiological conditions in the heart. Therefore, elevated generation of these factors may represent a potential compensatory response of cardiac cells to the cardiotoxic effects induced by 2-MCPD in rats.

Substantiating this hypothesis, elevated levels of proteins involved in maintaining myocardial integrity and function, such as Capg, Des, F-actin-capping protein subunit beta (Capzb), MICOS complex subunit Mic60 (Mic60), tubulin alpha-1A chain (Tuba1a), and EH domain-containing protein 1 (Ehd1) [48,49,50,51], were also observed in the 90-day oral toxicity rat study in Fischer F344/CDF rats exposed to 10 or 40 mg 2-MCPD/kg bw/day (Table 1). Moreover, a dose-dependent increase in structure- and/or function-maintaining proteins, myosin-binding protein C (Mybpc3) and myosin-7 (Myh7) (at 10 and 40 mg/kg bw/day), as well as alpha-actinin-1 (Actn1) (at 2, 10, and 40 mg/kg bw/day) [52,53,54], was found in rats orally exposed to 2-MCPD for 90 days (Table 1, Supplemental Figure S1).

Together, these data substantiate the hypothesis that oral exposure of rats to 2-MCPD leads to cardiotoxic effects in rats, which is reflected by the deregulation of structure-maintaining proteins, such as Actc1. Moreover, these effects induce compensatory processes, mediated via increased availability of cardioprotective proteins, such as Des and Wdr1, facilitating the maintenance of structural and functional integrity of the heart.

### 3.2. Mechanistic Implications Regarding 2-MCPD Biotransformation, Mitochondrial Metabolism and Function in the Heart

In 1979, Jones and Fakhouri postulated the loss of chlorine from 2-MCPD to yield an epoxide intermediate that would be cleared via glutathionylation to a mercapturic acid [17]. Although this pathway is chemically disfavored at physiologic pH, enzymatic catalysis to the epoxide is not yet disproven. However, about 40 years later, Bergau and colleagues experimentally proved, for the first time, the existence of considerable amounts of chlorinated 2-MCPD-metabolites in human urine, such as 2-chloro-3-hydroxy-propanoic acid (referred to as 2-chloro-hydracrylic acid or 2-ClHA) [55]. The authors postulated the intermediate formation of 2-chloro-3-hydroxy-propane aldehyde [55]. Although such metabolites may be formed predominantly in the liver, it is not yet disproven that this metabolic pathway involving alcohol and aldehyde dehydrogenases might be active in the heart to some extent as well. Targeted heart metabolism studies are so far missing. However, results of the reconsideration of our previously published in vivo proteomics data [19,20] suggest that there might be an association with additional metabolic pathways in the heart, such as pyruvate metabolism, generation of redox equivalents, and ATP synthesis (e.g., mitochondrial pyruvate dehydrogenase E1 component subunit alpha, creatine kinase S-type, and ATP synthase subunit alpha (Atp5f1a) and beta; see Table 1 and Supplemental Figure S1). This—in turn—could affect the high energy demand of cardiomyocytes and other cardiac cells in order to maintain cardiac functions.

### 3.3. Enzymes Utilizing or Producing Acetyl-Coenzyme A Necessary for Pyruvate Metabolism and Fatty Acid Degradation

One of the strongest dysregulated proteins found in this category was the mitochondrial acetyl-CoA acetyltransferase (Acat1) [56]. It was decreased in 2-MCPD-treated rats of the 28-day oral toxicity study as well as in animals of all dose groups of the 90-day study (see Table 1). Moreover, Ingenuity Pathway Analysis (IPA) of data from the 90-day study at 2 and 10 mg/kg bw/day showed that Acat1 is associated with functional networks in the rat heart (Supplemental Figure S1). This enzyme is involved in many metabolic pathways, such as acetyl-coenzyme A (CoA) biosynthesis, acetyl-CoA catabolism, fatty acid beta-oxidation, isoleucine catabolism, ketone body degradation, and propionyl-CoA biosynthesis, amongst others [57,58].

Also downregulated in the 90-day study at 10 and 40 mg 2-MCPD/kg bw/day, but not in the 28-day study, was mitochondrial 3-ketoacyl-CoA thiolase (Acaa2, Table 1). This enzyme catalyzes the last step of the mitochondrial beta-oxidation pathway, an aerobic process that breaks down fatty acids into acetyl-CoA. Using free CoA, this enzyme catalyzes the thiolytic cleavage of medium- to long-chain unbranched 3-oxoacyl-CoAs into acetyl-CoA and a fatty acyl-CoA shortened by two carbon atoms. It also catalyzes the condensation of two acetyl-CoA molecules into acetoacetyl-CoA and could be involved in the production of ketone bodies [59].

Trifunctional enzyme subunit alpha (Hadha), also known as monolysocardiolipin acyltransferase, includes long-chain enoyl-CoA hydratase and the long-chain 3-hydroxyacyl-CoA dehydrogenase [60] and was found to be downregulated in all dose groups of the 90-day study and the 10 mg/kg bw/day group of the 28-day study (Table 1). The mitochondrial trifunctional enzyme catalyzes the last three reactions of the mitochondrial beta-oxidation pathway, which is the major energy-producing process in the heart, based on the breakdown of fatty acids to acetyl-CoA. Among the enzymes involved in this pathway, the trifunctional enzyme exhibits specificity for long-chain fatty acids. Mitochondrial trifunctional enzyme is a heterotetrameric complex composed of two proteins: the trifunctional enzyme subunit alpha/HADHA, which carries the 2,3-enoyl-CoA hydratase and the 3-hydroxyacyl-CoA dehydrogenase activities, and the trifunctional enzyme subunit beta/HADHB, which bears the 3-oxoacyl-CoA thiolase activity. Hadha also exhibits a cardiolipin acyltransferase activity that participates in cardiolipin remodeling.

The mitochondrial succinyl-CoA:3-ketoacid coenzyme A transferase 1 was found to be upregulated only in the 28-day oral toxicity study at the 10 mg/kg bw/day dose (Table 1). This enzyme plays a key role in ketone body catabolism [61]. It catalyzes the first, rate-limiting step of ketone body utilization in extrahepatic tissues by transferring CoA from a donor thiolester species (succinyl-CoA) to an acceptor carboxylate (acetoacetate), leading to the generation of acetoacetyl-CoA. Acetoacetyl-CoA is further metabolized by acetoacetyl-CoA thiolase into two acetyl-CoA molecules, which enter the citric acid cycle for energy production.

The mitochondrial pyruvate carboxylase was not affected in the 28-day study but tended to be decreased in the lowest and highest dose groups of the 90-day study (Table 1). This enzyme catalyzes a two-step reaction involving the ATP-dependent carboxylation of the covalently attached biotin in the first step and the transfer of the carboxyl group to pyruvate in the second [62]. This leads to the formation of oxaloacetate, which then reacts with acetyl-CoA formed by pyruvate dehydrogenase complexes to yield citric acid via citrate synthase.

A highly deregulated enzyme following 2-MCPD treatment in rat hearts was the mitochondrial pyruvate dehydrogenase E1 component subunit alpha (Pdha1). It was downregulated in the 28-day study and in the lowest and highest dose groups of the 90-day study (Table 1). The pyruvate dehydrogenase complexes utilize the cofactors thiamine pyrophosphate, CoA, dihydrolipoamide, and nicotinamide adenine dinucleotide (NADH) for the production of acetyl-CoA from pyruvate and NAD+ in the mitochondrial matrix. The E1 subunit initially binds pyruvate and thiamine pyrophosphate [63,64]. Besides this, another protein subunit of pyruvate dehydrogenase, called E2, involved in the biological function of this complex, was also dysregulated in some dose groups. In the higher-dose groups of the 90-day study, mitochondrial dihydrolipoyl dehydrogenase (Dld) was upregulated. On the other hand, pyruvate dehydrogenase complex component E2 was dysregulated in the 28-day study and in the higher-dose groups of the 90-day study (Table 1).

The 2-oxoglutarate dehydrogenase component E1 (Ogdh) of the 2-oxoglutarate dehydrogenase complex was upregulated only in the 28-day gavage study (see Table 1). It participates in the first, rate-limiting step for the overall conversion of 2-oxoglutarate to succinyl-CoA and CO_2_, mainly in mitochondria. This enzyme catalyzes the irreversible decarboxylation of 2-oxoglutarate (alpha-ketoglutarate) via the thiamine diphosphate (ThDP) cofactor and subsequent transfer of the decarboxylated acyl intermediate on an oxidized dihydrolipoyl group that is covalently amidated to the E2 enzyme (dihydrolipoyllysine-residue succinyltransferase or DLST). It plays a key role in the citric acid cycle, which is a common pathway for oxidation of fuel molecules, including carbohydrates, fatty acids, and amino acids, and can be inhibited by superoxide and nitric oxide in vitro [65].

The 2-oxoisovalerate dehydrogenase subunit alpha (Bckdha) was upregulated in the 28-day oral toxicity study and in the highest dose of the 90-day oral toxicity study (Table 1). This subunit is also named branched-chain alpha-keto acid dehydrogenase E1 component alpha chain and forms, together with branched-chain keto acid dehydrogenase beta, the heterotetrameric E1 subunit of the mitochondrial branched-chain alpha-ketoacid dehydrogenase complex. This complex catalyzes the multi-step oxidative decarboxylation of alpha-ketoacids derived from the branched-chain amino acids valine, leucine, and isoleucine, producing CO_2_ and acyl-CoA, which is subsequently utilized to produce energy [66,67]. The E1 subunit catalyzes the first step with the decarboxylation of the α-ketoacid, forming an enzyme-product intermediate. A reductive acylation mediated by the lipoylamide cofactor of protein subunit E2 normally extracts the acyl group from the E1 active site for the next step of the reaction.

In the 28-day study, the mitochondrial glutamate dehydrogenase 1 (Glud1) that converts L-glutamate into α-ketoglutarate was markedly downregulated in the 28-day study (Table 1). This enzyme plays a key role in glutamine anaplerosis by producing alpha-ketoglutarate, an important intermediate in the citric acid cycle [68].

**Figure 2:**
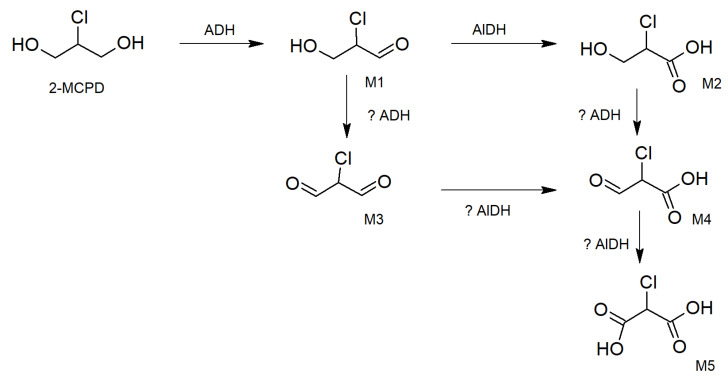
Putative metabolic pathways that might lead to 2-chloro-malonic acid as an intermediate in 2-MCPD metabolism. Metabolites of 2-MCPD postulated and found so far in human urine (M1 and M2; [55]), and putative metabolites thereof (M3, M4, and M5), such as 2-chloro-malonic semi- (M3) or dialdehydes (M4), or 2-chloro-malonic acid (M5), that might interfere with cellular energy supply. Metabolic enzymes: alcohol dehydrogenase (ADH) and aldehyde dehydrogenase (AlDH). “?” means that the involvement of this enzyme in the metabolism of 2-MCPD needs to be verified.

Interestingly, mitochondrial acetylating methylmalonate semialdehyde/malonate semialdehyde dehydrogenase (Aldh6a1), an A1 member of the aldehyde dehydrogenase family 6, was increased in the 28-day study and in the two highest dose groups of the 90-day oral toxicity study (Table 1). Whether this was due to increased semialdehyde concentrations from 2-MCPD (Figure 2) or a response to the need to funnel increased propionyl-CoA concentrations into the citric acid cycle remains speculative. If alcohol dehydrogenases had formed toxic semialdehydes in proximity or even inside mitochondria of cardiomyocytes, then mitochondrial proteins might have become compromised. This enzyme is also responsible for beta-alanine catabolism, a thymine catabolic process, and valine catabolism, amongst others [69].

A prominent protein annotated to the mitochondrial ATP synthase subunit alpha, ATP synthase subunit alpha (Atp5f1a) [70], was downregulated in the 28-day study and in the two higher-dose groups of the 90-day study (Table 1). This enzyme is linked to proton motive force-driven mitochondrial ATP synthesis, response to muscle activity, cellular response to glucocorticoids, cellular response to nitric oxide, lipid metabolic processes, and endothelial cell responses, amongst others [71,72,73]. The decrease in this enzyme may indicate a functional loss of ATP supply and/or concomitant loss of mitochondria in severely affected cells. If this were to happen in cardiomyocytes, irreversible heart failure may ensue.

The mitochondrial creatine kinase S-type (Ckmt2), a cardiolipin-binding protein [74], was found to be downregulated in the 28-day study only (Table 1). It reversibly catalyzes the transfer of phosphate between ATP and various phosphagens (e.g., creatine phosphate). Creatine kinase isoenzymes play a central role in energy transduction in tissues with large, fluctuating energy demands, such as skeletal muscle, heart, brain and spermatozoa [75].

### 3.4. Hypothesis of Reactive Intermediates Formed from 2-MCPD in Rat Heart

Based on recent findings of Bergau and colleagues [55] that 2-MCPD is metabolized 2-chlorohydracrylic acid (M2), most probably via formation of the intermediate to 2-chloro-3-hydroxy-propane aldehyde (M1) we postulate additional chlorinated metabolites (Figure 2). We speculate that from the aldehyde, a dialdehyde might be intermediarily formed by a second oxidation of the monoaldehyde (Figure 2). This putative metabolite may be the 2-chloro-propane 1,3-dial, also named 2-chloro-malondialdehyde (M3). As such, aldehydes are reactive towards glutathione, proteins, and DNA; their intracellular half-lives are deemed to be short. The depletion of intracellular glutathione and the oxidation of thioredoxin-1 may partially account for DNA damage-independent cytotoxicity of aldehydes. Cell survival against weakly toxic aldehydes is proposed to be dependent on DNA repair, whereas that against highly toxic aldehydes is deemed independent of DNA repair. If the amount of highly toxic aldehydes exceeds the scavenging capacity of cytosolic glutathione (GSH) and cysteine-containing proteins, a DNA damage-dependent cytotoxic effect could arise [76]. The 2-chloro-malondialdehyde (M3) may further be oxidized, e.g., by alcohol dehydrogenases, to the 2-chloro-malonic acid-semialdehyde, also called 2-chloro-formyl acetic acid (M4; [77]). Further oxidation, e.g., by aldehyde dehydrogenases, may then yield the 2-chloro-malonic acid (M5). The formation of those predicted metabolites M3, M4, and M5 in the liver and heart or other organs following 2-MCPD exposure remains to be experimentally investigated. Non-chlorinated malonic acid can normally be degraded to acetic acid and CO_2_ [78] and thus is hardly detectable in urine. Only in situations of sustained malonic acid decarboxylase inhibition or deficiency can malonic acid be easily detected in human urine [79]. Currently, it remains to be proven whether 2-chloro-malonic acid is formed in the human organism and whether it also resists decarboxylation to 2-chloro-acetic acid [78]. Only then would 2-chloro-malonic acid become detectable in urine.

Since there are many intracellular possibilities to trap the putative aldehydic intermediates by nucleophilic reactions, the identification of their reaction products, such as protein adducts or small molecule conjugates (e.g., with glutathione), is analytically very challenging, although it might become feasible in metabolically competent cellular test systems in vitro. In urine, the respective mercapturic acids, if formed in vivo, might be found at least in trace amounts.

**Figure 3:**
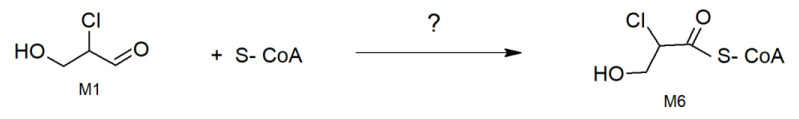
An example of a putative conjugate of 2-chlorinated 3-hydroxy-propionic aldehyde with CoA (M6). “?” means that such types of reactions of 2-MCPD-aldehydic intermediates with CoA need to be verified.

Even more challenging would be the detection of putative intermediates, such as CoA conjugated with chlorinated propionic aldehydes and acids (Figure 3). Those putative compounds might interfere with the normal C3 and C2 metabolism at the levels of dehydrogenases and decarboxylases and eventually result in altered metabolic patterns involving CoA moieties as cofactors in enzymatic reactions that normally regulate fatty acid degradation in the heart.

It appears as if CoA modifications are signals involved in the assembly or the degradation processes of distinct mitochondrial matrix proteins, such as acetyl-CoA acetyltransferase and 3-oxoacyl-CoA thiolase [80]. If such processes were disturbed by chlorinated molecules, these could compromise normal amino acid and/or fatty acid catabolism in heart tissues, such as delta(3,5)-delta(2,4)-dienoyl-CoA isomerase, mitochondrial (Ech1) [81].

Since malonic acid has long been known to interfere with energy metabolism [82], it appears reasonable to assume that 2-chlorinated semi- or dialdehydes, as well as 2-chlorinated propionic acid semialdehydes, and/or 2-chloro-malonic acid, if formed in vivo, might also become problematic to mitochondrial energy supply. They may become non-physiologic substrates for different enzymes involved in carbohydrate metabolism, including SCoA-catalyzed reactions in enzyme complexes, such as acetyl-CoA-, succinyl-CoA-, and/or methylmalonyl-CoA-mediated reactions [83]. Some of the enzymes involved might be propionyl-CoA carboxylase, pyruvate dehydrogenase, alpha-ketoglutarate dehydrogenase, and succinate dehydrogenase, as already reflected in Section 2 (see Table 1).

The results published by Bergau and colleagues [55] and our hypotheses regarding the putative formation of further reactive intermediates of 2-MCPD via alcohol and aldehyde dehydrogenases (see Figure 2 and Figure 3) led us to reinvestigate the proteomic data obtained from two earlier in vivo oral 2-MCPD toxicity studies in rats [19,20].

### 3.5. Summary

In the 28-day rat study using daily doses of 10 mg/kg bw/day 2-MCPD or 3-MCPD, or an equimolar (53 mg/kg bw/day) or a lower (13.3 mg/kg bw/day) dose of 2-MCPD-dipalmitate, comprehensive comparative proteomic analyses of substance-induced alterations in the heart were performed. These experiments revealed striking similarities between effects induced by 2-MCPD and its dipalmitate ester, whereas the degree of effect- overlap between 2-MCPD and 3-MCPD was much less [19]. The differentially deregulated proteins from rat hearts following 2-MCPD administration only marginally overlap with those found following 3-MCPD administration. Moreover, enzymes of the glycolytic pathways might be less affected by 2-MCPD than by 3-MCPD. This set of data demonstrates that even if they exert effects in the same organ and target similar metabolic networks, profound differences between the molecular effects of 2-MCPD and 3-MCPD exist. Therefore, it seems necessary to perform separate risk assessments for the two substances.

We found that daily repeated oral administration of 10 mg 2-MCPD/kg bw for 28 days via gavage led to distinct alterations in protein expression of some enzymes that might be directly involved in, or altered as a consequence of, the metabolism of 2-MCPD in rat heart cells. Interestingly, several prominently altered gene products are associated with dehydrogenases linked to glycolytic pathways and the citric acid cycle, such as the 2-oxoglutarate dehydrogenase component E1, the mitochondrial pyruvate dehydrogenase E1 component subunit alpha, and the trifunctional enzyme subunit alpha (see Table 1). These enzymes are important for enabling cellular energy production by fostering catabolic processes and delivering reducing equivalents and ATP to the various energy-dependent cell functions.

Moreover, we found altered expression patterns of some proteins, such as the mitochondrial ATP synthase subunit alpha and the mitochondrial creatine kinase S-type, that are connected to the cardiac carbohydrate and fatty acid turnover and consequently to the generation of redox equivalents (e.g., NADH) and ATP. From the data selected for hypothesis-driven discussion, it is tempting to assume that some of the putative metabolites, postulated above (see Figure 2 and Figure 3), might be responsible for cellular reactions leading to altered expressions and/or degradation of key mitochondrial enzymes needed for energy supply by using pyruvate or fatty acids as main fuels for oxidative phosphorylation.

Our findings regarding the abovementioned differentially regulated proteins point to complex interference of the metabolites of 2-MCPD with glycolytic and mitochondrial metabolic processes in the rat heart at a dose of 10 mg 2-MCPD/kg bw/day (as used in the 28-day gavage study [19]).

## 4. Materials and Methods

### 4.1. Data Sources/Animal Studies

The comparative analysis done here was based on data obtained from a subacute (28-day) oral toxicity study done with male Wistar rats (Fraunhofer ITEM Study No. 02 N 13 51) and a subchronic (90-day) oral toxicity study performed with male Fischer F344/CDF rats (ACC No. 2017-008/Pathology No. 187P). The studies were conducted according to the Organization for Economic Co-operation and Development (OECD) test guideline (TG) 407 (“Repeated Dose 28-day Oral Toxicity Study in Rodents”) and OECD TG 408 (“Repeated Dose 90-Day Oral Toxicity Study in Rodents”), respectively. Animals were orally exposed to 2-MCPD. In the 28-day rat study, 10 mg/kg body weight (bw) 2-MCPD was emulsified in corn oil and applied via gavage [19]. In the 90-day rat study, 2-MCPD was also applied orally [20]. More detailed information regarding the study design was published elsewhere [19,20,22]. Table 2 shows key data of the subacute (28-day) study and the subchronic (90-day) study done with rats orally exposed to 2-MCPD.

### 4.2. Proteomic Analysis

Proteomic studies were done via 2-D gel electrophoresis (2-DE) based on the methods provided by Goerg and colleagues [84]. Details on the 2-DE method were published elsewhere [19,20,85].

### 4.3. Comparative Analyses of the 28- and 90-Day Studies

#### 4.3.1. Evaluation and Characterization of Deregulated Protein Spots

Gel spot values were subjected to statistical evaluation when detected in gel images of 3 out of 5 animals (28-day study) or 4 out of 6 animals (90-day study) and 2 out of 4 gel replicates. Only spots deregulated at *p* ≤ 0.05 (Wilcoxon rank sum test) and a log_2_ ratio of 0.5 were included in further analysis.

#### 4.3.2. Gel Spot Identification

The procedures for protein isolation, analysis and identification are described in detail in [19,20,85].

#### 4.3.3. Data Interpretation

Ingenuity Pathway Analysis (IPA; https://digitalinsights.qiagen.com, QIAGEN, Hilden, Germany) was used to facilitate bioinformatic evaluation of differentially expressed proteins. Different proteoforms of the same protein identity, detected as distinct differentially expressed protein spots on 2-DE gels, could not be chemically characterized by the MALDI-MS/MS approach used in the proteomic studies. For this reason, only proteoforms showing opposing deregulation were subjected to bioinformatic analysis. For traceability, each of the most deregulated spots was selected. For the complete list of differentially expressed protein spots uploaded, refer to Supplemental Table S1. Based on the results of IPA, network analyses were done for all 2-MCPD dose groups included in the current comparative analyses of the proteomic results from the two aforementioned rat studies [19,20].

## 5. Conclusions

At the current state of knowledge, we cannot identify a clear mechanism of toxicity for 2-MCPD in mitochondria. However, the results found and presented here highlight that 2-MCPD, or merely some of its metabolites, might severely interfere with various pathways of cytosolic and mitochondrial carbohydrate, fatty acid, and amino acid metabolism. The adverse outcome of “heart failure” might result from 2-MCPD intermediates interfering with different pathways responsible for ATP formation that the heart tissue cannot compensate for through higher fatty acid degradation or glycolysis. Therefore, we hypothesize that the cardiotoxicity of 2-MCPD, possibly induced by some of its aldehydic and/or acidic metabolites, may be mediated—at least in part—via mitochondrial inhibition of energy supply in the rat heart. Furthermore, the dysregulated enzymes affecting the maintenance of cardiac structure and function are a strong indication of muscle-selective tissue disruption in rat hearts.

Some of these effects may already start at a dose of 2 mg/kg bw/day in the case of subchronic exposure and may be, to some extent, reversible. However, our current focused analysis of proteomic data from rat hearts following administration of moderate oral doses of 2-MCPD strengthens the weight of evidence that the impairment of mitochondrial functions could be related to adverse outcomes leading to cardiotoxicity and cardiomyocyte senescence.

Therefore, in the future, research should aim to falsify or verify the hypothesis presented here through further exposure time- and dose-dependent, tissue type-selective studies on mitochondrial energy metabolism. Those studies should also take into account the effects of 2-MCPD on the hemodynamic level in vivo. This is necessary to unveil the molecular initiating and key events of the adverse outcome pathway underlying the toxic actions of 2-MCPD in the heart. This kind of silently impaired functionality and viability of cardiomyocytes, a cell type that is not regenerated in adulthood in case of cell loss, calls for continuous control and mitigation of not only 3-MCPD but also 2-MCPD in human diets.

## Figures and Tables

**Table 1:** List of differentially expressed proteins mentioned in the text. Entries marked gray show opposing directions of deregulation in different spots “(-)” means that no significant difference regarding protein expression was found. For a comprehensive list of all identified proteins with more details, refer to Supplemental Table S2.

Gene Names	UniProt Entry	UniProt ID	Name(s) and Synonym(s) of the Protein	28-Day Study	90-Day Study		
				10 mg/kg bw	2 mg/kg bw	10 mg/kg bw	40 mg/kg bw
*Acaa2*	THIM_RAT	P13437	3-ketoacyl-CoA thiolase, mitochondrial	(-)	(-)	−0.84	−1.62
*Acat1*	THIL_RAT	P17764	Acetyl-CoA acetyltransferase, mitochondrial	(-)	1.09	1.37	1.23
*Acat1*	THIL_RAT	P17764	Acetyl-CoA acetyltransferase, mitochondrial	−3.19	−0.51	−1.15	−1.57
*Aco2*	ACON_RAT	Q9ER34	Aconitate hydratase, mitochondrial	1.05	(-)	(-)	(-)
*Actc1*	ACTC_RAT	P68035	Actin, alpha cardiac muscle 1	−0.77	(-)	−0.75	(-)
*Actn1*	ACTN1_RAT	Q9Z1P2	Alpha-actinin-1	(-)	1.17	1.59	1.55
*Aldh2*	ALDH2_RAT	P11884	Aldehyde dehydrogenase, mitochondrial	(-)	(-)	(-)	0.62
*Aldh6a1*	MMSA_RAT	Q02253	Methylmalonate semialdehyde, mitochondrial	0.81	(-)	0.63	0.69
*Aldh6a1*	MMSA_RAT	Q02253	Methylmalonate semialdehyde, mitochondrial	(-)	(-)	(-)	−1.03
*Atp5f1a*	ATPA_RAT	P15999	ATP synthase subunit alpha, mitochondrial	−0.74	(-)	−0.77	−0.85
*Atp5f1a*	ATPA_RAT	P15999	ATP synthase subunit alpha, mitochondrial	(-)	(-)	(-)	1.08
*Atp5f1b*	ATPB_RAT	P10719	ATP synthase subunit beta, mitochondrial	−0.67	(-)	(-)	−0.78
*Atp5f1c*	ATPG_RAT	P35435	ATP synthase subunit gamma, mitochondrial	(-)	(-)	(-)	0.67
*Bckdha*	ODBA_RAT	P11960	2-oxoisovalerate dehydrogenase subunit alpha, mitochondrial	0.77	(-)	(-)	0.78
*Capg*	CAPG_RAT	Q6AYC4	Macrophage-capping protein	0.75	(-)	(-)	0.86
*Capzb*	CAPZB_RAT	Q5XI32	F-actin-capping protein subunit beta	(-)	(-)	(-)	0.57
*Cav3*	Cav3_RAT	P51638	Caveolin-3	0.84	(-)	(-)	(-)
*Ckmt2*	KCRS_RAT	P09605	Creatine kinase S-type, mitochondrial	−0.56	(-)	(-)	(-)
*Des*	DESM_RAT	P48675	Desmin	0.81	(-)	(-)	0.82
*Dld*	DLDH_RAT	Q6P6R2	Dihydrolipoyl dehydrogenase, mitochondrial	(-)	(-)	1.16	1.61
*Ech1*	ECH1_RAT	Q62651	Delta(3,5)-Delta(2,4)-dienoyl-CoA isomerase, mitochondrial	0.55	(-)	(-)	0.57
*Eci1*	ECI1_RAT	P23965	Enoyl-CoA delta isomerase 1, mitochondrial	(-)	(-)	(-)	0.68
*Fh*	FUMH_RAT	P14408	Fumarate hydratase, mitochondrial	(-)	(-)	(-)	−0.60
*Glud1*	DHE3_RAT	P10860	Glutamate dehydrogenase 1, mitochondrial	−0.82	(-)	(-)	(-)
*Hadha*	ECHA_RAT	Q64428	Trifunctional enzyme subunit alpha, mitochondrial	−1.19	−0.88	−0.68	−0.73
*Hsp90b1*	ENPL_RAT	Q66HD0	Endoplasmin	(-)	(-)	(-)	0.58
*Hspa1a*	HS71A_RAT	P0DMW0	Heat shock 70 kDa protein 1A	(-)	(-)	(-)	0.69
*Hspa4*	HSP74_RAT	O88600	Heat shock 70 kDa protein 4	(-)	(-)	0.50	0.61
*Hspa5*	BIP_RAT	P06761	Endoplasmic reticulum chaperone BiP	(-)	(-)	(-)	0.87
*Hspb1*	HSPB1_RAT	P42930	Heat shock protein beta-1	0.93	(-)	(-)	0.54
*Mybpc3*	MYPC_RAT	P56741	Myosin-binding protein C, cardiac-type	(-)	(-)	0.80	2.62
*Myh7*	MYH7_RAT	P02564	Myosin-7 (Myosin heavy chain 7)	(-)	(-)	0.74	0.88
*Myl2*	MLRV_RAT	P08733	Myosin regulatory light chain 2, ventricular/cardiac muscle isoform	(-)	(-)	(-)	−0.50
*Ogdh*	ODO1_RAT	Q5XI78	2-oxoglutarate dehydrogenase complex component E1	0.64	(-)	(-)	(-)
*Oxct1*	SCOT1_RAT	B2GV06	Succinyl-CoA:3-ketoacid coenzyme A transferase 1, mitochondrial	0.71	(-)	(-)	1.12
*Pdha1*	ODPA_RAT	P26284	Pyruvate dehydrogenase E1 component subunit alpha, somatic form, mitochondrial	−0.98	−0.80	(-)	−0.61
*Pdk1*	PDK1_RAT	Q63065	[Pyruvate dehydrogenase (acetyl-transferring)] kinase isozyme 1, mitochondrial	−0.51	(-)	(-)	(-)
*Tpm1*	TPM1_RAT	P04692	Tropomyosin alpha-1 chain	1.04	(-)	(-)	(-)
*Tpm2*	TPM2_RAT	P58775	Tropomyosin beta chain	2.37	(-)	(-)	(-)
*Tpm3*	TPM3_RAT	Q63610	Tropomyosin alpha-3 chain	0.56	(-)	(-)	(-)
*Wdr1*	WDR1_RAT	Q5RKI0	WD repeat-containing protein 1	1.22	(-)	(-)	(-)

**Table 2:** Experimental details of rat studies used for proteomic analyses.

Experimental Details	28-Day Study	90-Day Study
according to OECD-Test No.	407 (subacute)	408 (subchronic)
species (strain)	rat (Wistar)	rat (Fischer F344/CDF)
sex and age	male weanlings	male and female weanlings
animal number	6	12/12
test substance supplier/Cat No.	2-MCPD TRC Toronto	2-MCPD TRC Toronto
administration	oral gavage	pellet diet
dose groups investigated by proteome analysis: mg/kg bw/day	0, 10	0, 2, 10, 40
vehicle	corn oil 7.5 mL/kg bw	corn oil, achieving 7% in the diet
diet	Ssniff V1534	AIN-93G
necropsy/anesthesia	Whole body perfusion/pentobarbital	Whole body perfusion/isoflurane

## Data Availability

The data presented in this study is available on request from the corresponding author (the data is not publicly available due to privacy).

## Supplementary Materials

The following supporting information can be downloaded at https://www.mdpi.com/article/10.3390/molecules31040692/s1, Figure S1: Results from Ingenuity Pathway Analysis; Table S1: Complete list of deregulated protein spots from rat heart after 28- and 90-day oral exposure to 2-MCPD; Table S2: List of deregulated proteins from rat heart after 28- and 90-day oral exposure to 2-MCPD. Opposite deregulation of the same protein in different spots is highlighted in gray (IPA-upload list); Table S3: IPA: toxicologically relevant functions; Table S4: IPA: pathway-related protein patterns; and Legends: Legends to Figure S1 and Tables S1–S4.
