# Supplementary material for: 2-MCPD-Induced Effects in the Heart: Toxicological and Mechanistic Implications from Comparative Proteomic Analyses in Rats
Source: Molecules. 2026 Feb 17;31(4):692. doi: 10.3390/molecules31040692 (PMC12943361; doi:10.3390/molecules31040692)
Supplement: Supplementary file 1 [file molecules-31-00692-s001.zip › Supplemental_tables_legends_molecules-4070882_v2.pdf]

Legend to supplemental tables

Figure S1	Results from Ingenuity Pathway Analysis: selected functional networks of all treatments associated to interactions of deregulated proteins following oral exposure to 2-MCPD.
Table S1	Complete list of identified deregulated protein spots from rat heart after 28 and 90 days oral exposure to 2-MCPD. Color code visualizes patterns of differentially expressed proteoforms of proteins. Please note that proteins are sorted according their UniProt entry names.
Table S2	Table S2 List of identified deregulated proteins from rat heart after 28 und 90 days oral exposure to 2-MCPD. Opposite deregulation of the same protein in different spots are highlighted in grey.
Table S3	Results from Ingenuity Pathway Analysis: list of toxicity categories and associated deregulated proteins in treatment groups. Functions present in Figure 1 are marked.
Table S4	Results from Ingenuity Pathway Analysis: list of deregulated proteins in treatment groups, which are associated to canonical pathways.
