# Supplementary figures and images for: 2-MCPD-Induced Effects in the Heart: Toxicological and Mechanistic Implications from Comparative Proteomic Analyses in Rats
Source: Molecules. 2026 Feb 17;31(4):692. doi: 10.3390/molecules31040692 (PMC12943361; doi:10.3390/molecules31040692)

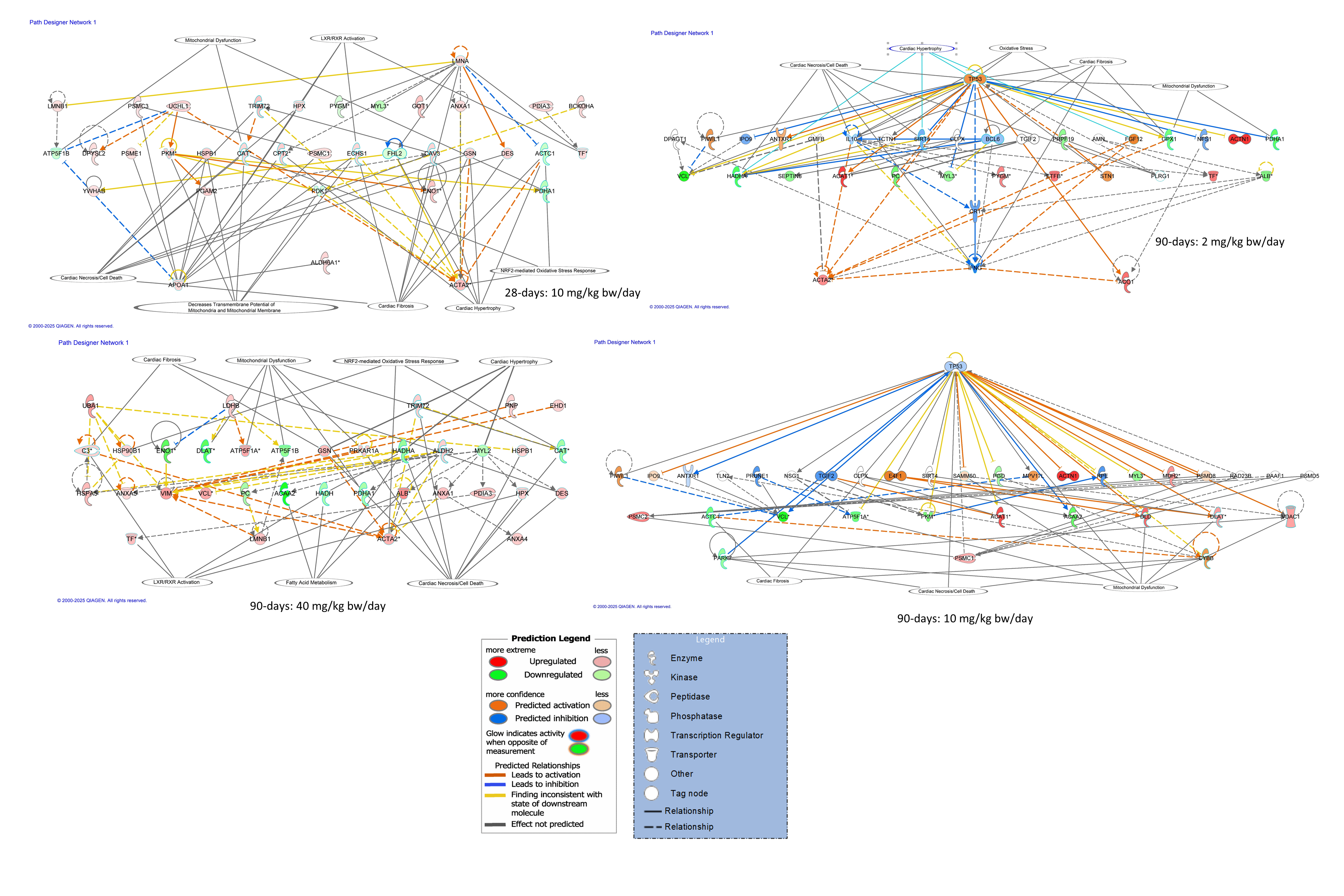

Supplement: Supplementary file 1 [file molecules-31-00692-s001.zip › Supplemental_Figure_S1_molecules-4070882_v2.tif]
